# Contrasting roles of fungal necromass and mineral preservation in influencing mineral-associated organic carbon across and within elevation bands

**DOI:** 10.3389/fmicb.2026.1897611

**Published:** 2026-07-30

**Authors:** Junyu Zhu, Qinxi Liu, Xuyang Lu, Jiasen Wu, Xinli Chen, Youchao Chen, Yanjiang Cai

**Affiliations:** 1 State Key Laboratory for Development and Utilization of Forest Food Resources, Zhejiang A&F University, Hangzhou, China; 2 College of Environment and Resources (College of Carbon Neutrality), Zhejiang A&F University, Hangzhou, China; 3 College of Carbon Neutrality, Zhejiang A&F University, Hangzhou, China; 4 Institute of Mountain Hazards and Environment, Chinese Academy of Sciences, Chengdu, China

**Keywords:** fungal residues, mineral preservation capacity, particulate organic carbon, soil carbon stabilization, soil texture, subtropical forest

## Abstract

Mineral-associated organic carbon (MAOC) is a persistent fraction of soil organic carbon (SOC) that is central to long-term carbon (C) persistence and climate mitigation. Along an elevation gradient, soil texture and chemistry vary with climate and coincide with shifts in microbial communities, but it remains unclear whether MAOC aligns more strongly with abiotic soil attributes or biotic microbial necromass, and whether these relationships change from across-gradient patterns to within-elevation variation. In this study, we investigated SOC fractions in a subtropical mountain system and examined the factors associated with MAOC variation across the elevation gradient and within elevation bands. Across the elevation gradient, MAOC variation was best explained by elevation-associated changes in soil mineral properties, including amorphous Fe oxide, clay + silt content and pH. In contrast, within individual elevation bands, MAOC was positively correlated with total microbial necromass carbon (TNC). In addition, fungal necromass carbon (FNC) explained more variation in MAOC than bacterial necromass carbon (BNC); and TNC was significantly correlated with fungal community composition within bands. Notably, the relationship between TNC and MAOC weakened with increasing elevation. Taken together, our results indicate that MAOC patterns along the elevation gradient are primarily linked to mineral preservation, whereas within-band variation in MAOC is more closely related to bulk-soil microbial necromass C. These findings may help to reconcile conflicting views on MAOC regulation and highlight the context-dependency of soil C stabilization.

## 1 Introduction

Soil serves as the largest organic carbon (C) reservoir in terrestrial ecosystems (Schmidt et al., 2011; Paula et al., 2022). The accrual and stabilization of soil organic C (SOC) can be better described if SOC is partitioned into particulate organic carbon (POC) and mineral-associated organic carbon (MAOC) (Cotrufo et al., 2019; Lavallee et al., 2020). The POC primarily consists of partly decomposed plant residues, whereas MAOC is mainly derived from low-molecular-weight organic compounds released from plants or produced through microbial transformation and subsequently stabilized via mineral associations (Lavallee et al., 2020). Globally, MAOC accounts for about 65% of SOC in mineral soils and turns over far more slowly than POC, making it a primary determinant of long-term SOC persistence (Sokol et al., 2022; Zhou et al., 2024). Yet, the processes and environmental factors that govern MAOC formation and accrual remain only partly resolved, limiting efforts to manage soils for enhanced C stabilization and climate mitigation.

The formation and stabilization of MAOC are determined by the interactions of organic inputs, microbial processing, and protection by soil minerals (Sokol et al., 2022). Recent frameworks suggest that microbial necromass is a primary precursor of MAOC, because microbial residues have high affinity for mineral surfaces and can be immobilized via various stabilization mechanisms (e.g., sorption and cation bridging) (Liang et al., 2017, 2019). For example, incubation experiments and global syntheses indicate that microbial residues are an important constituent of stable soil organic matter (Kallenbach et al., 2016) and microbial necromass C can constitute a large proportion of MAOC (Liang et al., 2019). However, emerging studies suggest that soil minerals can be as important as microorganisms in increasing SOC persistence and stabilization (e.g., Xiao et al., 2023). The capacity of soil to stabilize C ultimately relies on organo-mineral associations, which are regulated by soil mineral properties (Schweizer et al., 2021; Rodríguez-Albarracín et al., 2023). Among these, clay + silt content, pH, and Fe oxides (e.g., amorphous Fe oxide: Feo) are widely recognized as robust proxies for mineral protection capacity. Specifically, clay and silt fractions provide the physical surface area for organic C adsorption (Schweizer et al., 2021); pH modulates the chemical bonding environment and mineral reactivity (Rowley et al., 2018); and Feo stabilizes organic C through ligand exchange, inner-sphere complexation, and coprecipitation with dissolved organic matter (Jia et al., 2024). Recent cross-site evidence has highlighted that mineral preservation capacity overrides microbial residue production in governing MAOC formation and stabilization along a weathering gradient (Zhu et al., 2024). Taken together, these findings suggest that MAOC is jointly regulated by microbial necromass input and soil mineral properties, but the dominant control is unlikely to be universal and may instead vary across environmental contexts and within-site conditions (Mei et al., 2025).

At regional to global scales, where climate and soil mineral attributes vary widely, MAOC concentration is often best predicted by soil mineral properties related to mineral protection, while vegetation and microbial variables usually play indirect roles (Georgiou et al., 2022; Rodríguez-Albarracín et al., 2023; Zhou et al., 2024; Zhu et al., 2024). In contrast, within a given ecosystem, where climate and mineral properties are relatively homogeneous, local variability in plant inputs, microbial community composition and microbial necromass C can explain a substantial part of the variation in SOC and MAOC fractions (Kallenbach et al., 2016; Yang et al., 2025). Nevertheless, most previous work has been conducted either across broad climatic gradients or at plot level within single sites, and few studies have simultaneously examined these controls by integrating both scales within the same system. This leaves a key gap in our understanding of whether, and how, the dominant control on MAOC differs between abiotic regulation (e.g., climate and soil mineral properties) across broad environmental gradients and biotic regulation (e.g., microbial necromass) within relatively comparable edaphic conditions. Addressing this gap is crucial because it helps us to generalize when microbial necromass serves as an effective precursor of MAOC vs. when mineral attributes impose a capacity constraint on MAOC stabilization.

Elevation gradients in mountains offer an ideal “natural laboratory” to address this question. Elevation gradients show distinct variation in climate, vegetation, and soil development over relatively short geographic distances, while maintaining within-band heterogeneity in vegetation and microbial communities within each elevation band (Hu et al., 2019; Mou et al., 2021; Zhang et al., 2025). This allows for the simultaneous assessment of how soil properties related to mineral protection (e.g., Feo, clay + silt content and pH) and microbial inputs (e.g., total microbial necromass C: TNC) regulate MAOC across an elevation gradient and within elevation bands. In this study, we conducted a field investigation in a subtropical mountain system spanning four forest types along a 700–1600 m elevation gradient. We examined SOC and its fractions (POC and MAOC), key soil properties (including clay + silt content, pH, and Feo), vegetation productivity (kNDVI), microbial community composition and necromass C across and within elevation bands. Specifically, we aimed to (1) examine elevational patterns of SOC fractions and associated environmental variables; (2) identify the major abiotic and biotic factors associated with MAOC variation across the elevation gradient and within elevation bands. We hypothesized that, across the elevation gradient, MAOC would be primarily controlled by soil mineral properties, whereas TNC would play a minor or indirect role. In contrast, we expected that within each elevation band, microbial necromass C would be more important than soil properties in influencing MAOC, reflecting the importance of microbial necromass inputs under relatively similar environmental background.

## 2 Materials and methods

### 2.1 Site description

The study area is located in Baishanzu, Qingyuan County, Lishui City, Zhejiang Province, eastern China (27°32′25.47″-29°27′19.79″N, 118°03′59.06″-119°22′09.18″E), with elevations ranging from 291 to 1929 m. The region is characterized by a mid-subtropical warm and humid climate. The bedrock is dominated by Jurassic igneous rocks, and the soils are mainly classified as yellow soils according to the Chinese Soil Taxonomy, which roughly correlate with Alisols in the World Reference Base system. It should be noted that while yellow soil is the dominant taxonomic unit across the study area, this classification encompasses pedological variation in clay mineralogy, organic matter content, and weathering intensity that is expected along an elevation gradient. Along the elevation gradient, four elevation bands were selected (700–800 m, 1100–1300 m, 1400–1500 m, and 1500–1600 m). Within each elevation band, we identified areas with similar slope and aspect and established twelve 20 m × 20 m plots spaced at least 50 m apart. Basic site characteristics for the plots at each elevation band are summarized in Table 1.

**Table 1:** Site information for the plots at each elevation band.

Elevation gradient	Longitude and latitude	Vegetation type	Dominant vegetation	Average tree height/m	Average tree diameter at breast height (DBH)/cm	MAT/°C	MAP/mm
700–800 m	119°1′13.2″E ~ 119°1′29.2″E 27°40′53.7″N ~ 27°41′27.8″N	Broadleaved forest	*Schima superba* Gardner and Champ.; *Castanopsis eyrei* (Champ. ex Benth.) Tutcher; *Castanopsis carlesii* (Hemsl.) Hayata.	7.6	11.3	17.2	1,872
1100–1300 m	119°10′29.6″E ~ 119°11′43.9″E 27°41′19.7″N ~ 27°44′2.4″N	Needle-broadleaved forest	*Cunninghamia lanceolata* (Lamb.) Hook.; *Pinus hwangshanensis* W. Y. Hsia; *Schima superba* Gardner and Champ.	11.2	11.4	12.1	2,012
1400–1500 m	119°11′33.4″E ~ 119°11′42.4″E 27°42′26.2″N ~ 27°42′41.3″N	Needle forest	*Pinus hwangshanensis* W. Y. Hsia	8.5	25.4	11.2	2,018
1500–1600 m	119°11′51.2″E ~ 119°11′57.6″E 27°45′12.9″N ~ 27°45′29.3″N	Scrub forest	*Rhododendron fortunei* Lindl.; *Eurya rubiginosa var. attenuata* H.T.Chang; *Eurya hebeclados* Ling	5.1	3.8	10.5	2,123

The average tree height and diameter at breast height (DBH) were calculated based on field measurements of the woody individuals. Mean annual temperature (MAT) and mean annual precipitation (MAP) for each elevation band were extracted from the ERA5-Land dataset based on the geographic coordinates of the plots.

### 2.2 Field investigation and sampling

Soil sampling was conducted in August 2023, coinciding with the peak growing season when soil temperature and moisture are generally favorable for microbial activity. In each plot, five soil cores (0–20 cm depth) were collected after surface litter was carefully removed. Prior to soil collection, a 1 m × 1 m quadrat was established in each plot to harvest all surface litter, which was then oven-dried to constant weight to determine litter standing stock. The five soil cores were then thoroughly mixed to obtain one composite sample. Our sampling approach yielded 48 composite samples in total. All soil samples were passed through a 2 mm sieve to remove visible roots and stones and then divided into subsamples for different analyses. One subsample was immediately frozen at −80 °C for DNA extraction and subsequent characterization of bacterial and fungal community composition. Another subsample was air-dried at room temperature and used to determine SOC and its fractions (POC and MAOC), microbial necromass C, and soil mineral properties including pH, fine mineral fraction (clay + silt), and Feo.

### 2.3 Sample analysis

#### 2.3.1 Kernel normalized difference vegetation index

To characterize plot-level vegetation productivity, we used the kernel normalized difference vegetation index (kNDVI), which has been shown to better capture vegetation dynamics and productivity in terrestrial ecosystems than conventional NDVI (Camps-Valls et al., 2021). Following Meng et al. (2024), we computed kNDVI using the Google Earth Engine (GEE) platform based on Sentinel-2 images for the Baishanzu region from 2017 to 2023. For each year, all available images were filtered by acquisition date and cloud cover, and clouds and cloud shadows were masked using the quality assessment bands. Annual mean kNDVI layers at 10 m spatial resolution were then generated for the study area (Supplementary Figure S1). Finally, using the geographic coordinates of each plot, we extracted the corresponding kNDVI values from the 2017–2023 mean kNDVI product.

#### 2.3.2 Soil properties and physical fractionation

Soil pH was determined with a pH meter (Mettler Toledo, USA) in a 1:2.5 (w/v) soil-to-deionized water suspension. Soil texture was determined using the hydrometer method (Kettler et al., 2001), and the sum of clay and silt was used as an index of the fine mineral fraction (clay + silt content) in subsequent analyses. Feo was determined following the acid ammonium oxalate method (McKeague and Day, 1966). Briefly, 0.1–0.5 g of air-dried soil (< 0.25 mm) was extracted with 10 mL of 0.2 mol L^−1^ ammonium oxalate buffer (pH 3.2) at a soil-to-solution ratio of 1:100. The suspension was shaken for 4 h at 25 °C in the dark and then centrifuged. An aliquot of the supernatant was reduced with hydroxylamine hydrochloride and reacted with 1,10-phenanthroline, and Fe concentration was measured colorimetrically at 510 nm.

Bulk soils were physically fractionated to separate POC and MAOC fractions following previous work (Cotrufo et al., 2019). Briefly, 20 g of air-dried soil passed through a 2 mm sieve was weighed into an Erlenmeyer flask, and 60 mL of 0.5% (w/v) sodium hexametaphosphate solution was added. The suspension was shaken for 18 h at 25 °C and 180 r·min^−1^, and then wet-sieved through a 53 μm nylon mesh, with the residue rinsed with distilled water until the effluent ran clear. The coarse fraction (particle size > 53 μm) was defined as particulate organic matter, and the fine fraction (particle size < 53 μm) as mineral-associated organic matter. The separated samples were oven-dried at 60 °C and gently ground. Both fractions were then sieved through 0.15 mm sieve prior to the measurement of organic C concentration using an elemental analyzer (VarioMAX C/N; Elementar, Germany).

Concentrations of glucosamine (GluN) and muramic acid (MurA) in bulk soil were determined using gas chromatography (GC; Agilent 7820, Agilent Technologies, USA). Briefly, soil samples were hydrolyzed with 6 mol L^−1^ HCl, spiked with 100 μg of inositol, and then filtered and purified. The hydrolysates were adjusted to neutral pH and evaporated to dryness, and amino sugars were extracted with anhydrous methanol and converted to aldononitrile acetate derivatives. Using inositol as an internal standard, 200 μL of an n-hexane:ethyl acetate mixture (1:1, v/v) was added to extract the derivatized products, which were subsequently separated and quantified by GC. The bacterial necromass C (BNC) and fungal necromass C (FNC) were calculated according to the following formula (Hu et al., 2024):


$$\begin{array}{lll} \mathrm{BNC} & = & MurA\times 31.3\\ \mathrm{FNC} & = & \left(GluN-1.16\times MurA\right)\times 10.8 \end{array}$$

where 31.3 and 10.8 are the conversion factors. The TNC is the sum of BNC and FNC.

#### 2.3.3 DNA extraction and processing

Bulk soil samples from each plot were used to characterize bacterial and fungal communities. DNA was extracted using the DNeasy PowerSoil Pro Kit (Qiagen, Hilden, Germany) according to the manufacturer's instructions. For each soil sample, two parallel extractions were performed and the DNA extracts were pooled prior to downstream analyses. The pooled DNA was then split for amplification of the bacterial 16S rRNA gene (V3–V4 region) and the fungal ITS1 region with universal primer sets.The amplicons were barcoded, purified and quantified, and sequencing was carried out by Novogene (Beijing, China) on an Illumina NovaSeq 6000 platform. Raw sequence reads were demultiplexed and subjected to quality control following Novogene's standard pipeline. High-quality sequences were processed in QIIME2 (version 2022.02) for downstream bioinformatic analysis. Amplicon sequence variants (ASVs) were inferred using the DADA2 pipeline. Bacterial ASVs were taxonomically assigned against the SILVA database release 138.1, whereas fungal ASVs were annotated using the UNITE database version 9.0. The resulting ASV tables were rarefied to an even sequencing depth before downstream analyses, with 47,513 reads per sample for the bacterial 16S rRNA dataset and 50,336 reads per sample for the fungal ITS dataset. These thresholds were lower than the minimum number of non-chimeric reads among the samples retained for this study. Rarefaction curves were provided in the Supplementary Figure S2). Chao1, Shannon, and Simpson diversity indices for both bacterial and fungal communities were calculated using the *vegan* package in R. Fungal functional groups were further inferred from the ITS1 ASVs using the FUNGuild database (accessed on 27 September, 2023). To reduce assignment uncertainty, only ASVs assigned with “probable” or “highly probable” confidence were retained for guild-level analysis. This filtering retained 2,768 of 13,641 ITS ASVs (20.29%) and 866,771 of 2,416,128 ITS reads (35.87%).

### 2.4 Statistical analysis

All statistical analyses were conducted using R software (version 4.0.5; http://www.r-project.org/). One-way analysis of variance (ANOVA) followed by Duncan's test was applied to examine differences in SOC fractions and related variables among elevation bands (*agricolae* package). Simple linear regression analyses were performed to evaluate relationships between MAOC and potential controlling factors across the elevation gradient. Structural equation modeling (SEM) was implemented to explore the hypothesized pathways linking climatic conditions, soil properties, vegetation, and MAOC along the elevation gradient (*lavaan* package). In the SEM, climatic conditions were represented by aridity index (AI), calculated following Xu et al. (2024) as AI = MAP / (MAT + 10). Soil properties were characterized by soil pH, clay + silt content, and Feo, and vegetation was represented by kNDVI. All 48 plots were used as observational units for the SEM because MAOC, soil properties, and vegetation variables were measured independently at the plot level. However, the climate variable represented an elevation-band-level gradient and therefore had four unique values, one for each elevation band. Prior to SEM analysis, multicollinearity among the environmental variables included in the model was assessed using variance inflation factors (VIFs). All VIF values were below 5 (Supplementary Table S1), indicating no severe multicollinearity among candidate predictors. Within each elevation band, bivariate linear regressions were used to examine pairwise associations between MAOC and each candidate predictor. To test whether the slope of the MAOC-TNC relationship differed among elevation bands, we performed an analysis of covariance (ANCOVA) with MAOC as the response variable, TNC as a continuous covariate, elevation band as a categorical factor, and the TNC × elevation-band interaction. A significant interaction term indicates that the strength of the MAOC-TNC relationship varies among elevation bands. The relative importance of BNC and FNC on MAOC was assessed using the *calc.relimp* function in the *relaimpo* package based on the Lindeman-Merenda-Gold (LMG) method. Microbial community structure was quantified using principal coordinate analysis (PCoA) based on Bray-Curtis dissimilarities calculated from the rarefied bacterial 16S rRNA and fungal ITS ASV tables. Bray-Curtis distance matrices were first generated using the *vegan* package in R, and PCoA was then performed separately for the bacterial and fungal communities. The first two PCoA axes (PCoA1 and PCoA2), which represented the major gradients in community composition, were extracted for subsequent analyses. Within each elevation band, Spearman correlation analyses were used to examine the associations between TNC and the bacterial or fungal PCoA axes. In addition, linear regression analyses were performed within each elevation band to examine the relationships between TNC and the relative abundances of predicted fungal functional guilds derived from FUNGuild.

## 3 Results

### 3.1 Elevational patterns of SOC fractions and associated environmental variables

**Figure 1:**
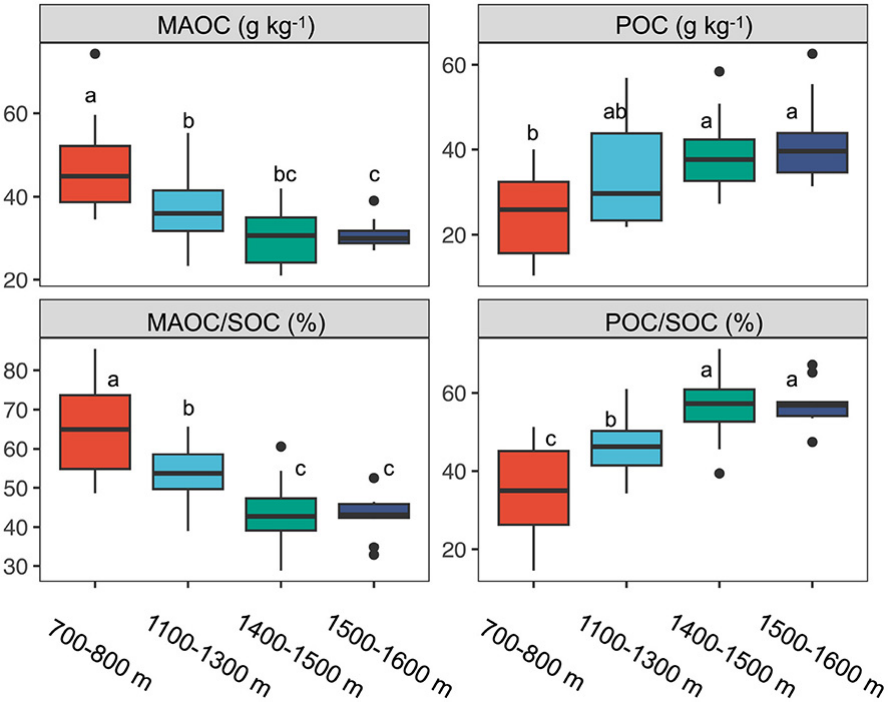
Elevational patterns of soil organic carbon fractions. POC, particulate organic carbon; MAOC, mineral-associated organic carbon. Different low case letters indicate a significant difference among elevation bands (*p* < 0.05). Data are shown for 12 plots per elevation band (*n* = 12).

MAOC declined significantly from 47.31 g kg^−1^ at 700–800 m (Broadleaved forest) to 30.95 g kg^−1^ at 1500–1600 m (Scrub forest). In contrast, POC generally showed an increasing trend along the elevational gradient, with the lowest value at 700–800 m (25.34 g kg^−1^) and the highest value at 1500–1600 m (41.68 g kg^−1^). Consequently, the MAOC/SOC ratio decreased from 65.25% at 700–800 m to 43.12% at 1500–1600 m, whereas the POC/SOC ratio increased from 34.74% to 56.88% (Figure 1).

**Figure 2:**
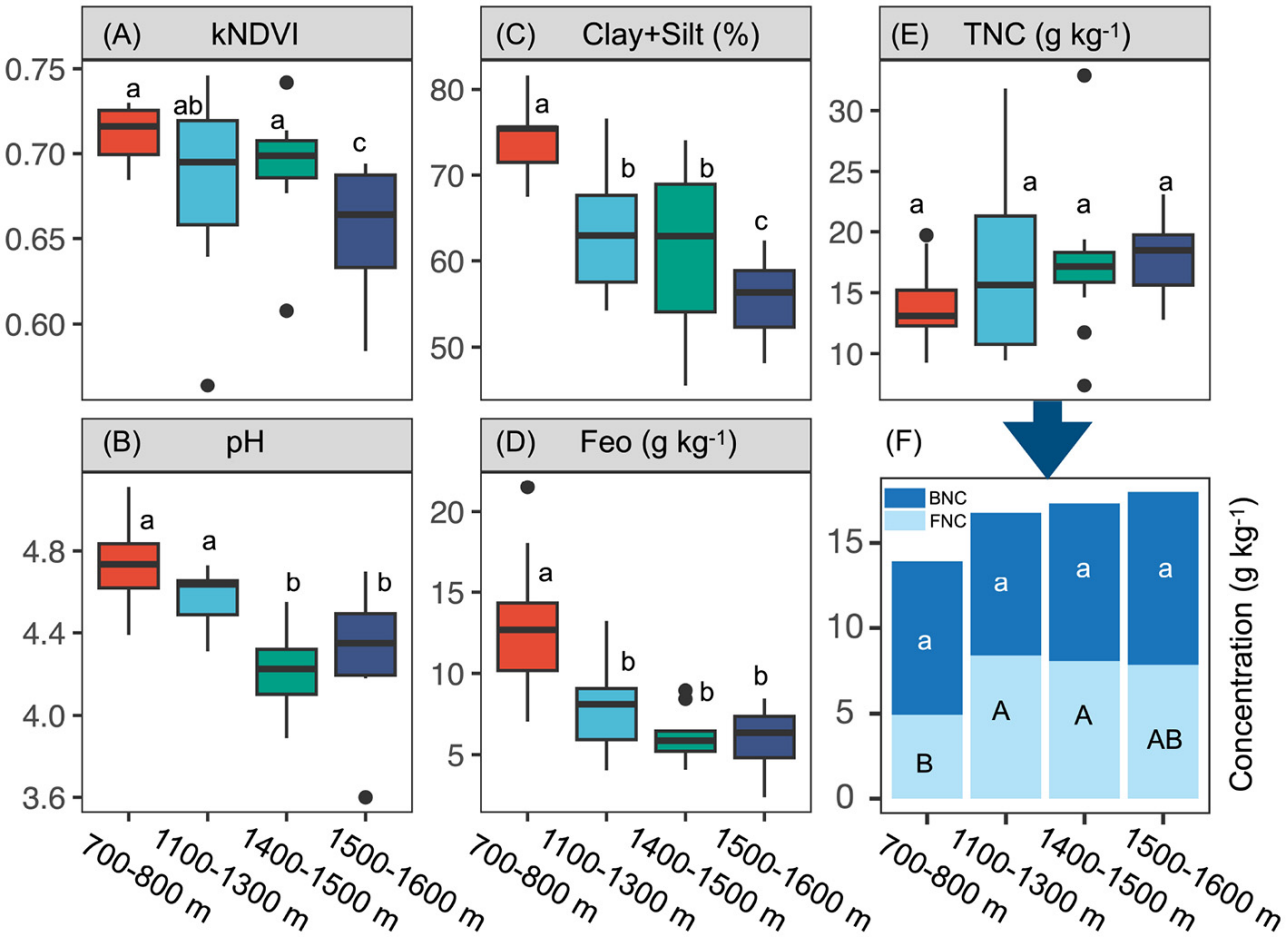
Elevational patterns of the potential drivers of mineral-associated organic carbon (MAOC). **(A)** kNDVI, kernel normalized difference vegetation index; **(B)** pH, soil pH; **(C)** Clay + Silt, total content of soil clay and silt; **(D)** Feo, amorphous Fe oxide; **(E)** TNC, total necromass C; **(F)** BNC, bacterial necromass C, and FNC, fungal necromass C. Different low and upper case letters indicate a significant difference among elevation bands (*p* < 0.05). Data are shown for 12 plots per elevation band (*n* = 12).

The changes in SOC fractions coincided with distinct elevational trends in key environmental variables (Figure 2). kNDVI decreased from 0.71 at 700–800 m to 0.66 at 1500–1600 m. Soil clay + silt content also declined significantly, from 74.50% to 55.48% across the gradient, and soil pH decreased from 4.73 to 4.32. Amorphous iron oxides (Feo) showed a significant decline from 12.74 g kg^−1^ at 700–800 m to 5.92 g kg^−1^ at 1500–1600 m. In contrast, TNC showed no significant differences among elevations, remaining within 13.94~17.99 g kg^−1^ across elevation gradient. Generally, BNC accounted for a higher proportion of TNC than FNC across elevation gradient, particularly at 700–800 m and 1500–1600 m (Figure 2F). BNC showed no significant differences among elevations, whereas FNC exhibited modest variation, with significantly higher values at mid-elevations than at 700–800 m.

### 3.2 Factors associated with MAOC variation across the elevational gradient

**Figure 3:**
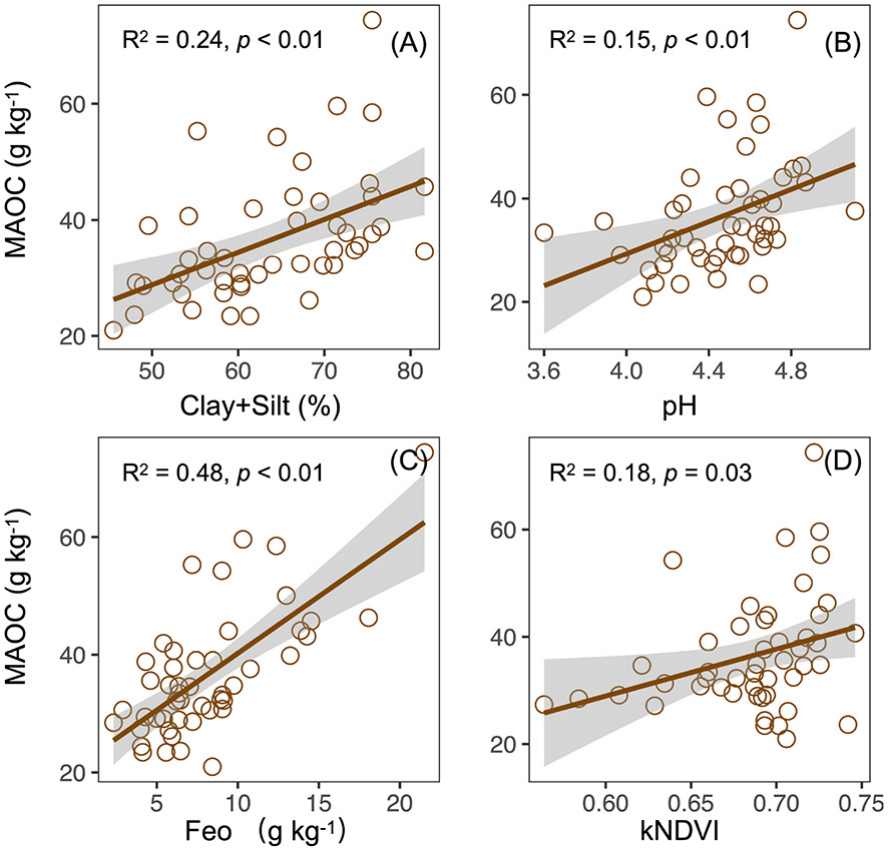
Relationships between mineral-associated organic carbon (MAOC) and the candidate predictors across elevation gradient. **(A)** Clay + Silt, total content of soil clay and silt; **(B)** pH, soil pH; **(C)** Feo, amorphous Fe oxide; **(D)** kNDVI, kernel normalized difference vegetation index.

**Figure 4:**
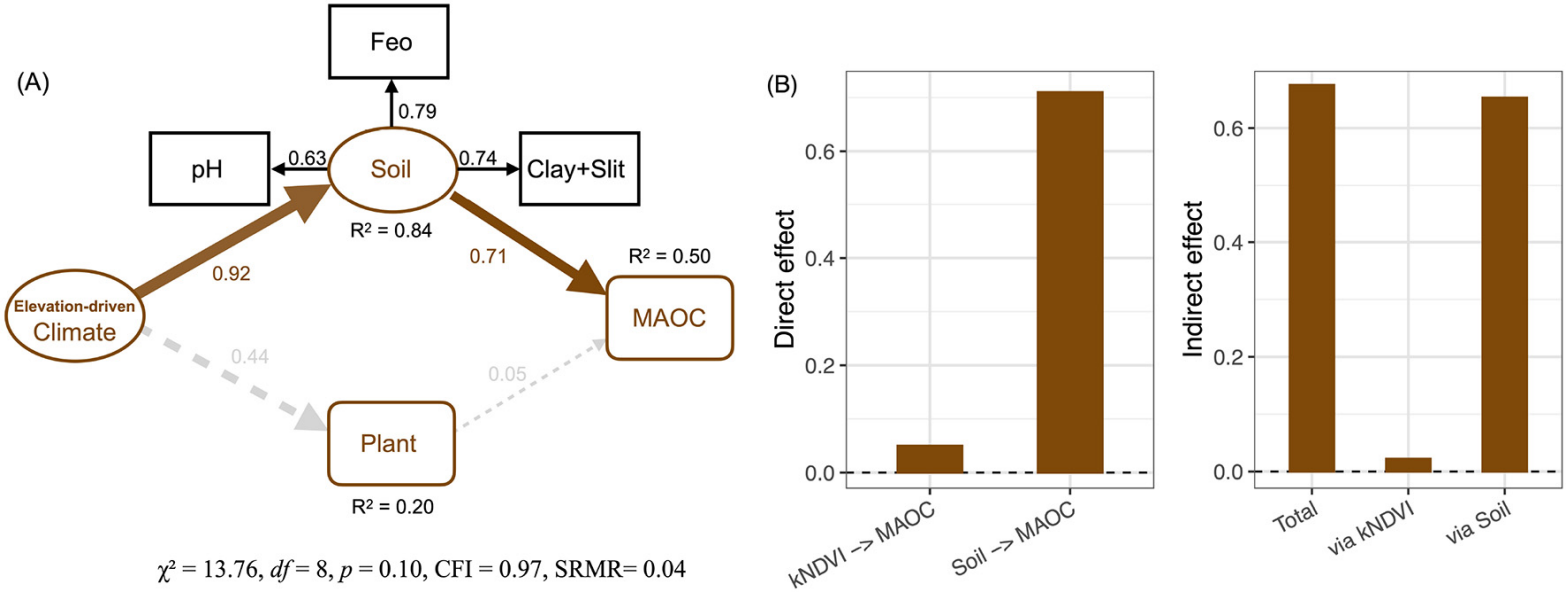
Structural equation model (SEM) illustrating the potential pathway by which climate, soil properties, and vegetation influence mineral-associated organic carbon (MAOC) **(A)**. Climate is represented as aridity index (AI), soil properties (Soil) as a latent variable composed of soil pH, clay + silt, and amorphous Fe oxide (Feo) content, and vegetation (Plant) as the kernel normalized difference vegetation index (kNDVI). Solid arrows indicate significant standardized paths (*p* < 0.05), whereas gray dashed arrows denote non-significant paths. Numbers adjacent to arrows represent standardized path coefficients, and values above response variables indicate the proportion of explained variance (*R*^2^). **(B)** The direct effects and indirect effects on MAOC.

Across the elevation gradient, MAOC was positively correlated with soil clay + silt content (*R*^2^ = 0.24, *p* < 0.01), soil pH (*R*^2^ = 0.15, *p* < 0.01), Feo (*R*^2^ = 0.48, *p* < 0.01) and kNDVI (*R*^2^ = 0.18, *p* = 0.03) (Figure 3), but did not show significant relationships with TNC, BNC or FNC (Supplementary Figure S3). The SEM results showed that the hypothesized pathways are highly consistent with the observed covariance structure underlying MAOC variation (χ^2^ = 13.76, *df* = 8, *p* = 0.10, CFI = 0.97, SRMR = 0.04) (Figure 4A). Climatic conditions (AI) along the elevation gradient significantly influenced soil properties (standardized path coefficient = 0.92), which in turn exerted a dominant control on MAOC (0.71). In contrast, the pathway from climate to vegetation (kNDVI) was weak (0.44), and vegetation had only a non-significant effect on MAOC (0.05). Partitioning of effects further revealed that soil properties accounted for most of the direct influence on MAOC, while vegetation contributed only a small fraction. The indirect effect of climate on MAOC occurred mostly through its regulation of soil properties, whereas the climate → vegetation → MAOC pathway was negligible (Figure 4B and Supplementary Table S2).

### 3.3 Elevation-specific associations with MAOC within each elevation band

**Figure 5:**
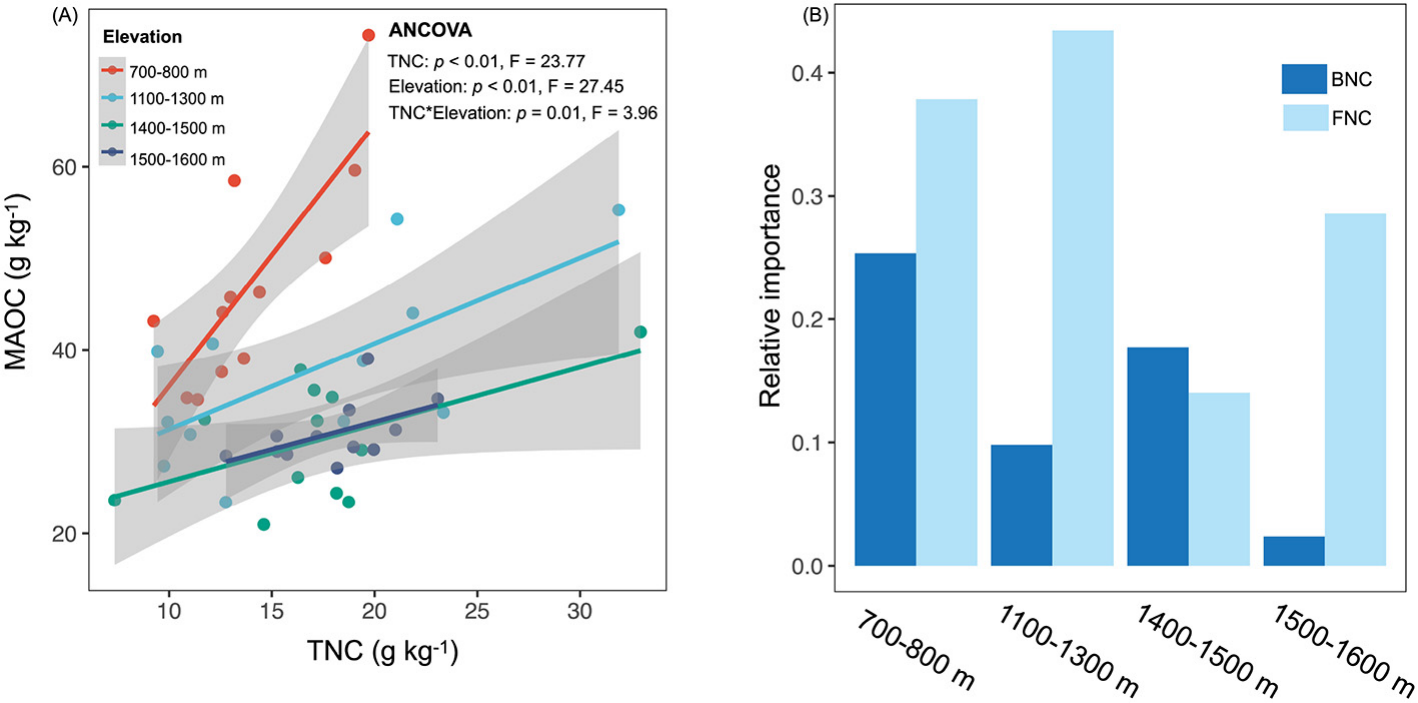
Elevation-specific relationships between microbial necromass carbon and MAOC. In panel **(A)**, analysis of covariance (ANCOVA) revealed significant effects of total microbial necromass carbon (TNC), elevation, and their interaction (TNC × Elevation). In panel **(B)**, relative explanatory contributions of bacterial necromass carbon (BNC) and fungal necromass carbon (FNC) to MAOC variation within each elevation band was calculated using Lindeman-Merenda-Gold (LMG) method. Regressions in panel **(A)** and variable-importance estimates in panel **(B)** were both based on 12 plots per elevation band (*n* = 12).

Within each elevation band, the MAOC–TNC relationship was positive but weakened markedly with increasing elevation (Figure 5A and Supplementary Table S3). Both the slope and explanatory power were highest at 700–800 m (slope = 2.86, *R*^2^ = 0.62, *p* < 0.01) and declined monotonically toward higher elevations: 1100–1300 m (slope = 0.94, *R*^2^ = 0.44, *p* = 0.02), 1400–1500 m (slope = 0.62, *R*^2^ = 0.35, *p* = 0.05), and 1500–1600 m (slope = 0.60, *R*^2^ = 0.29, *p* = 0.06). FNC generally covaried more strongly with MAOC than BNC within elevation bands (Figure 5B). Overall, BNC was positively correlated with MAOC within most elevation bands, but its explanatory power was generally lower and less consistent than that of FNC (Supplementary Table S3). The bivariate associations between MAOC and individual mineral or vegetation-related variables further suggested that mineral-related variables (e.g., Feo and Clay + silt) also showed significant associations with MAOC at higher elevations (Supplementary Figure S4).

**Figure 6:**
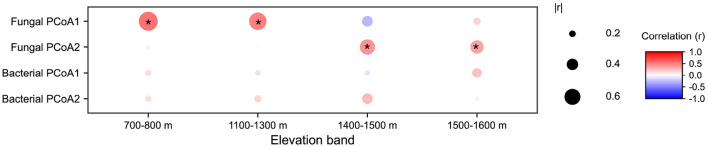
Correlations between TNC and the first two bacterial and fungal principal coordinate analysis (PCoA) axes within each elevation band. Bubble color indicates Spearman's correlation coefficient (*r*), and bubble size represents the absolute correlation strength (|*r*|). Asterisks indicate significant correlations (*p* < 0.05).

To explore microbial associations these elevation-specific TNC-MAOC relationships, we further examined how TNC was linked to microbial diversity (Supplementary Figures S5, S6) and community composition (Figure 6 and Supplementary Figure S7) within each gradient band. Spearman correlations showed that TNC was significantly related to fungal community composition at all elevation bands (Figure 6). Consistent with this pattern, functional guild analysis revealed significant positive relationships between TNC and the relative abundance of specific mycorrhizal and saprotrophic fungi (Supplementary Figure S8; all *R*^2^ ≥ 0.38, *p* < 0.05). However, because only 35.87% of ITS reads were retained after confidence filtering, and these sequencing data reflect the standing community at a single sampling time, the guild-level patterns should be interpreted with caution.

## 4 Discussion

**Figure 7:**
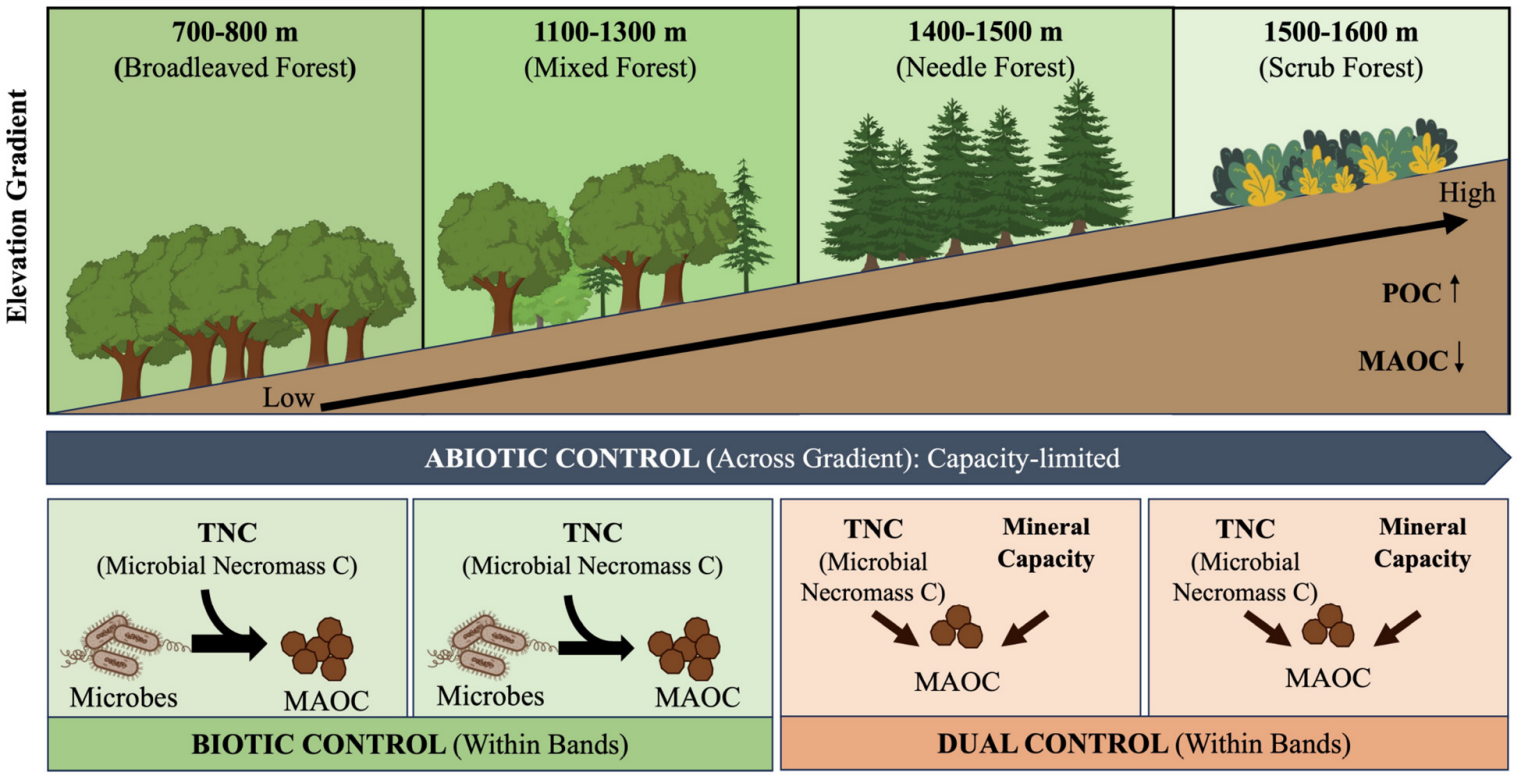
Conceptual framework illustrating the factors associated with MAOC variation across the elevation gradient and within elevation bands.

Although recent works have highlighted the critical role of microbial necromass and mineral protection in stabilizing MAOC (Liang et al., 2019; Xiao et al., 2023), empirical evidence disentangling their relative importance across environmental gradients remains scarce. Here, our study provided field-based evidence that the dominant correlate of MAOC variation shifted from mineral preservation capacity across the whole elevation gradient to microbial necromass supply within individual elevation bands. Specifically, our results revealed that elevation-driven climatic differences were associated with changes in soil mineral properties (clay + silt content, pH, and Feo), which appeared to constrain the MAOC concentration across the 700–1600 m elevation gradient; while TNC was the main correlate of MAOC variability within individual elevation bands where the edaphic background is relatively comparable. This finding helps to reconcile conflicting views in current studies about the influencing factors on MAOC, suggesting that the dominant correlate of MAOC is context-dependent along the elevation gradient (Figure 7).

### 4.1 Mineral preservation, rather than microbial necromass, is the dominant correlate of MAOC across elevation gradient

Our study showed that soil MAOC decreased significantly with increasing elevation (Figure 1), and it also decreased with the reduction in soil clay + silt content, pH and Feo across the whole 700–1600 m gradient (Figures 2, 3). The elevational decline in these three soil properties can be attributed to climatic constraints. At higher elevations, lower temperatures likely inhibit chemical weathering rates, thereby limiting the formation of secondary clay minerals and poorly crystalline iron oxyhydroxides (Riebe et al., 2004); concurrently, intense leaching from high precipitation and organic acids released from coniferous litter likely reduce base cations, resulting in soil acidification at higher elevations (Smith et al., 2002). Consistent with these mechanisms, the SEM indicated that elevation-driven climate (AI) strongly shaped soil mineral properties, which in turn exerted the dominant direct influence of MAOC (Figure 4). In contrast, although MAOC was positively correlated with kNDVI in the bivariate analysis, the climate –> vegetation –> MAOC pathway was weak and non-significant in the SEM after accounting for soil mineral controls (Figures 3, 4). In the present study, the decline in the fine mineral fraction (clay + silt) from low to high elevations likely reduced mineral specific surface area and reactive sites, thereby constraining organo-mineral bonding and limiting the formation of MAOC (Hassink, 1997; Schweizer et al., 2021). The concurrent reduction in Feo further diminished the availability of reactive mineral phases that stabilize organic compounds through ligand exchange and coprecipitation (Jia et al., 2024). Furthermore, the reduction in soil pH at high elevations likely weakened cation bridging (Rowley et al., 2018) or enhanced aluminum toxicity (Jones et al., 2019), further limiting C stabilization. These results are consistent with the view that, when comparing sites across broad environmental gradients, MAOC concentration is primarily associated with soil mineral attributes such as clay and silt content (Zhu et al., 2024), reactive metal oxides (e.g., Feo), and associated chemical properties (e.g., pH) (Zhou et al., 2024; Liu et al., 2026).

Interestingly, we did not observe significant relationships between MAOC and microbial necromass C across the elevation gradient (Supplementary Figure S3). Meanwhile, TNC remained relatively stable among elevations (Figure 2E). Together, these results also indicated that, across the elevation gradient, MAOC variation was not primarily constrained by the supply of microbial residues, but rather by the soil's mineral stabilization capacity. This interpretation is consistent with the concept of soil C saturation (Georgiou et al., 2025; Liu et al., 2025); thus, even if the microbes produce necromass C across elevations, the capacity of soil to stabilize these microbial residues into MAOC is ultimately constrained by the soil mineral attributes. A concentration-based C balance check further supports this interpretation. Total SOC did not differ significantly among elevation bands (69.5–72.7 g kg^−1^; one-way ANOVA, *p* = 0.93), yet MAOC declined by 16.4 g kg^−1^ from the lowest to the highest band while POC increased by 16.3 g kg^−1^. Because TNC did not decline along the gradient (Figure 2E), the increase in POC at high elevation is unlikely to reflect a decline in microbial necromass C. Instead, litter standing stock increased significantly with elevation (Supplementary Figure S9A) and was strongly and positively associated with POC (*R*^2^ = 0.48, *p* < 0.001; Supplementary Figure S9B). Together, these relationships indicate that, as mineral protection capacity weakened with elevation, C that could no longer be stabilized in the mineral-associated fraction accumulated instead as litter-derived POC. This pattern is further supported by a meta-analysis across Chinese forests (Cheng et al., 2025). However, we note that litter-derived C was inferred from litter standing stock and its association with POC rather than partitioned directly (e.g., by isotopic or biomarker tracing), and this attribution warrants confirmation in future work. We also note that vegetation type changed along the elevation gradient, from broadleaved forest to scrub forest. Thus, vegetation composition and litter quality likely covaried with elevation and may have contributed to the observed POC/MAOC patterns by altering litter decomposability and organic matter inputs. Because vegetation type was not experimentally separated from elevation, its effects cannot be fully disentangled from climate and soil mineral changes in this study.

### 4.2 Microbial necromass supply is associated with MAOC within elevation bands

In contrast to the whole-gradient pattern, significant positive correlations between TNC and MAOC were observed within each elevation band (Figure 5A). The TNC-MAOC relationship within elevation bands supports the Microbial Carbon Pump (MCP) framework, which emphasizes that microbial anabolism and the subsequent necromass production can contribute to the stabilization of SOC (Liang et al., 2017). This result implies that the MCP mechanism could dominate the MAOC formation under conditions where climate, vegetation, and the mineralogical properties are relatively similar. In addition, our results showed that FNC generally explained more variations in MAOC than BNC (Figure 5B and Supplementary Table S3), suggesting that fungal-mediated pathways may be more important for MAOC formation within elevation bands. This pattern does not contradict the larger contribution of BNC to the total necromass pool, because higher pool size does not necessarily equate to higher spatial explanatory power. Rather, our results indicate that FNC covaried more strongly with plot-level MAOC variation, even when BNC represented a larger standing pool in some elevation bands. A possible reason is that fungal necromass often contains complex and persistent cell-wall compounds (e.g., chitin and melanin), which exhibit slower degradation rates and higher stability and thus have a greater chance of being transferred into MAOC fraction (Cong et al., 2025; Gao et al., 2025).

Consistent with this interpretation, we also found that TNC was consistently related to fungal community composition within elevation bands (Figure 6). In addition, the exploratory FUNGuild analysis showed positive relationships between TNC and the relative abundance of selected fungal guilds within each elevation band (Supplementary Figure S8). For example, in the present study, arbuscular mycorrhizal fungi at low elevation may promote rapid microbial biomass turnover and necromass production under relatively favorable climatic and edaphic conditions, whereas ectomycorrhizal fungi at high elevation may contribute more recalcitrant, structurally complex necromass that can be stabilized in MAOC (Fernandez et al., 2019; Wang et al., 2019). These patterns suggest that, within individual elevation bands where environmental conditions are relatively homogeneous, variation in fungal community composition and predicted guild structure is associated with necromass C and, ultimately, MAOC (Beidler et al., 2025). However, these guild-level patterns should be interpreted with caution because high-confidence FUNGuild assignments (probable or highly probable) represented only 20.29% of ITS ASVs and 35.87% of ITS reads. In addition, fungal functional assignments were inferred from ITS1 ASVs using FUNGuild, and this approach is constrained by database coverage, assignment confidence, and primer-related biases. In particular, ITS primers may show variable performance across fungal functional groups, and ITS1 alone may not adequately capture some groups such as arbuscular mycorrhizal fungi, for which 18S rRNA sequencing is often recommended as a complementary marker (George et al., 2019). Furthermore, microbial necromass C integrates residue inputs over years to decades, whereas our sequencing data capture only a single-timepoint (August 2023) snapshot of the standing community. We therefore regard these fungal community- and guild-level results as exploratory evidence rather than direct functional measurements; validating them will require time-resolved sampling combined with complementary marker genes, functional assays, or direct tracing of necromass origin (e.g., isotopic or biomarker labeling). Interestingly, the relationship between TNC and MAOC weakened as increasing elevation (Figure 5A and Supplementary Table S3) and soil mineral properties emerging as significant correlates of MAOC (Supplementary Figure S4). The fading correlation likely reflects a decline in C stabilization efficiency rather than a simple lack of microbial necromass input. At low elevations, where soil clay + silt, pH and Feo were higher, the soil mineral matrix is sufficient to allow the microbial necromass to be immobilized into MAOC via strong organo-mineral associations. Conversely, at high elevations, even the microbial necromass C is high, the significantly lower clay + silt content and Feo concentration likely constrained MAOC formation through reduced mineral surface area and fewer reactive sorption sites. It should also be noted that microbial necromass C was quantified in bulk soil rather than directly in the MAOC fraction. Therefore, the observed TNC-MAOC relationships indicate a strong association between microbial necromass C and MAOC concentration, but they do not directly demonstrate the incorporation of microbial necromass into the MAOC fraction. Future studies combining physical fractionation with amino-sugar or isotopic analyses would be needed to directly trace microbial necromass allocation into MAOC.

## 5 Conclusion

By using a typical subtropical mountain elevation gradient (700–1600 m), we show that MAOC concentration cannot be explained by a single, uniform mechanism. When the entire elevation gradient is considered, changes in soil mineral properties—specifically clay+silt content, pH, and Feo—appeared to set the soil's capacity to stabilize organic C and was the dominant correlate of MAOC. In contrast, within individual elevation bands where environmental conditions were similar, microbial necromass C, especially FNC, was more closely associated with MAOC variation. In particular, the strength of microorganisms in explaining MAOC variation declined with the increased elevation, likely reflecting weaker mineral protection at higher elevations. These findings suggest that the dominant control on MAOC can shift between broad environmental gradients and within-site variation, which may help to reconcile seemingly divergent conclusions in the MAOC literature. We emphasize, however, that our study is restricted to strongly weathered, acidic subtropical forest soils (yellow soils / Alisols) developed on igneous parent material. Because the mineral-capacity limitation we describe likely depends strongly on parent material and weathering regime, these inferences should be extrapolated with caution to other pedogenic contexts. Validation in soils with contrasting mineralogy, such as calcareous or volcanic (andic) soils, is needed before this framework can be generalized.

## Data Availability

The datasets presented in this study can be found in online repositories. The names of the repository/repositories and accession number(s) can be found in the article/Supplementary material.
