# Supplementary material for: Contrasting roles of fungal necromass and mineral preservation in influencing mineral-associated organic carbon across and within elevation bands
Source: Front Microbiol. 2026 Jul 30;17:1897611. doi: 10.3389/fmicb.2026.1897611 (PMC13469646; doi:10.3389/fmicb.2026.1897611)
Supplement: Supplementary file 1 [file Table_1.docx]

Fig. S1 The kernel normalized difference vegetation index (kNDVI) of Baishanzu region

Fig. S2 Rarefaction curves for bacterial 16S rRNA (A) and fungal ITS (B) datasets. Light gray lines represent individual samples, colored lines represent elevation-band means, and shaded areas indicate ± SE. Dashed vertical lines indicate the rarefaction depths used for downstream analyses: 47,513 reads per sample for 16S and 50,336 reads per sample for ITS.

Table S1 Variance inflation factors (VIFs) for environmental variables used in the structural equation model

| **Predictor** | **VIF** |
| --- | --- |
| AI | 3.75 |
| kNDVI | 1.27 |
| pH | 1.65 |
| Clay+silt | 2.09 |
| Feo | 2.06 |

Note: VIF, variance inflation factor; AI, aridity index; kNDVI, kernel normalized difference vegetation index; Feo, amorphous Fe oxide. MAT and MAP were not included simultaneously with AI because AI was calculated from climatic variables.

Fig. S3 Relationship between mineral-associated organic carbon (MAOC) and total microbial necromass carbon (TNC), fungal necromass carbon (FNC), and bacterial necromass carbon (BNC)

Table S2 Standardized direct, indirect, and total effects from the SEM

| Response variable | Predictor | Effect type | Pathway | Standardized effect |
| --- | --- | --- | --- | --- |
| MAOC | Soil latent factor | Direct effect | Soil -> MAOC | 0.71 |
| MAOC | kNDVI | Direct effect | kNDVI -> MAOC | 0.05 |
| MAOC | Elevation-driven climate | Indirect effect | Climate -> Soil -> MAOC | 0.65 |
| MAOC | Elevation-driven climate | Indirect effect | Climate -> kNDVI -> MAOC | 0.02 |
| MAOC | Elevation-driven climate | Total indirect effect | Climate -> Soil/kNDVI -> MAOC | 0.68 |
| MAOC | Elevation-driven climate | Total effect | Direct + indirect effects | 0.68 |
| Soil latent factor | Elevation-driven climate | Direct effect | Climate -> Soil | 0.92 |
| kNDVI | Elevation-driven climate | Direct effect | Climate -> kNDVI | 0.44 |

Note: Values are standardized effects from the structural equation model. Soil is a latent factor represented by pH, clay+silt, and Feo.

Table S3 Linear regression showing the relationship between mineral-associated organic carbon (MAOC) and microbial necromass carbon within each elevation band

| Predictor | Elevation | Vegetation | Slope | SE | *p* value | R² |
| --- | --- | --- | --- | --- | --- | --- |
| TNC | 700-800 | Broadleaved forest | 2.86 | 0.35 | <0.01 | 0.62 |
|  | 1100-1300 | Needle-broadleaved forest | 0.94 | 0.17 | 0.02 | 0.44 |
|  | 1400-1500 | Needle forest | 0.62 | 0.15 | 0.05 | 0.35 |
|  | 1500-1600 | Scrub forest | 0.60 | 0.10 | 0.06 | 0.29 |
| BNC | 700-800 | Broadleaved forest | 5.34 | 2.32 | 0.04 | 0.35 |
|  | 1100-1300 | Needle-broadleaved forest | 1.82 | 1.25 | 0.18 | 0.18 |
|  | 1400-1500 | Needle forest | 1.12 | 0.56 | 0.07 | 0.28 |
|  | 1500-1600 | Scrub forest | 0.16 | 0.53 | 0.77 | 0.01 |
| FNC | 700-800 | Broadleaved forest | 3.10 | 1.04 | 0.01 | 0.47 |
|  | 1100-1300 | Needle-broadleaved forest | 1.36 | 0.42 | 0.01 | 0.51 |
|  | 1400-1500 | Needle forest | 0.98 | 0.54 | 0.10 | 0.25 |
|  | 1500-1600 | Scrub forest | 0.69 | 0.36 | 0.08 | 0.27 |

Note: TNC, total microbial necromass carbon; BNC, bacterial necromass carbon; FNC, fungal necromass carbon; MAOC, mineral-associated organic carbon. Each regression was fitted separately within each elevation band, with *n* = 12 per band.

Fig. S4 Relationships between MAOC concentration and candidate environmental predictors within each elevation band. Bivariate linear regressions were performed separately for each elevation band (n = 12 per band) to examine the associations of MAOC with Feo, clay+silt content, pH, and kNDVI. Solid brown lines indicate significant regressions (*p* < 0.05), whereas gray dashed lines indicate non-significant regressions

Fig. S5 Relationship between total microbial necromass carbon (TNC) and bacterial diversity for each elevation band.

Fig. S6 Relationship between total microbial necromass carbon (TNC) and fungal diversity for each elevation band. Solid brown lines indicate significant regressions (*p* < 0.05), whereas gray dashed lines indicate non-significant regression

Fig. S7 Microbial community composition across elevation bands by using principal coordinate analysis (PCoA) ordinations of bacterial 16S rRNA and fungal ITS communities based on Bray-Curtis dissimilarities. Points represent individual soil samples and colors/symbols indicate elevation bands.

Fig. S8 Relationship between the relative abundance of fungal functional guilds and total necromass C (TNC) within each elevation band. Each regression was fitted within each elevation band using 12 plots (n = 12).

Fig. S9 Litter standing stock (A) and its relationship with particulate organic carbon (POC) (B) across elevation gradient. Different low case letters indicate a significant difference among elevation bands (*p* < 0.05).
